# TRPV1 Tunes Optic Nerve Axon Excitability in Glaucoma

**DOI:** 10.3389/fphys.2020.00249

**Published:** 2020-03-26

**Authors:** Nolan R. McGrady, Michael L. Risner, Victoria Vest, David J. Calkins

**Affiliations:** Department of Ophthalmology and Visual Sciences, Vanderbilt Eye Institute, Vanderbilt University Medical Center, Nashville, TN, United States

**Keywords:** glaucoma, transient receptor potential vanilloid member 1, optic nerve, compound action potential, nodes of Ranvier, NaV1.6

## Abstract

The transient receptor potential vanilloid member 1 (TRPV1) in the central nervous system may contribute to homeostatic plasticity by regulating intracellular Ca^2+^, which becomes unbalanced in age-related neurodegenerative diseases, including Alzheimer’s and Huntington’s. Glaucomatous optic neuropathy – the world’s leading cause of irreversible blindness – involves progressive degeneration of retinal ganglion cell (RGC) axons in the optic nerve through sensitivity to stress related to intraocular pressure (IOP). In models of glaucoma, genetic deletion of TRPV1 (*Trpv1^–/–^*) accelerates RGC axonopathy in the optic projection, whereas TRPV1 activation modulates RGC membrane polarization. In continuation of these studies, here, we found that *Trpv1^–/–^* increases the compound action potential (CAP) of optic nerves subjected to short-term elevations in IOP. This IOP-induced increase in CAP was not directly due to TRPV1 channels in the optic nerve, because the TRPV1-selective antagonist iodoresiniferatoxin had no effect on the CAP for wild-type optic nerve. Rather, the enhanced CAP in *Trpv1^–/–^* optic nerve was associated with increased expression of the voltage-gated sodium channel subunit 1.6 (NaV1.6) in longer nodes of Ranvier within RGC axons, rendering *Trpv1^–/–^* optic nerve relatively insensitive to NaV1.6 antagonism via 4,9-anhydrotetrodotoxin. These results indicate that with short-term elevations in IOP, *Trpv1^–/–^* increases axon excitability through greater NaV1.6 localization within longer nodes. In neurodegenerative disease, native TRPV1 may tune NaV expression in neurons under stress to match excitability to available metabolic resources.

## Introduction

Transient receptor potential vanilloid member 1 (TRPV1) channels are activated by both physiologically relevant and pathological stimuli, conducting large Ca^2+^ currents that initiate downstream signaling cascades (Caterina et al., 1997; Hui et al., 2003; Patapoutian et al., 2009; Weitlauf et al., 2014). TRPV1 channels densely accumulate in nociceptor cells of dorsal root ganglia to transduce noxious sensory input into the electrochemical responses of the spinal nerve (Simone et al., 1989; Caterina et al., 1997, 2000; Bolcskei et al., 2005). Recent evidence shows widespread TRPV1 expression in the central nervous system (CNS) tissues, including the cortex, hippocampus, hypothalamus, and retina (Mezey et al., 2000; Roberts et al., 2004; Cristino et al., 2006; Sappington et al., 2009, 2015; Jo et al., 2017; Lakk et al., 2018). TRPV1 has also been implicated in neurodegenerative disorders such as Alzheimer’s disease (Jayant et al., 2016; Balleza-Tapia et al., 2018), Parkinson’s disease (Marinelli et al., 2003; Morgese et al., 2007; Nam et al., 2015; Chung et al., 2017), Huntington’s disease (Lastres-Becker et al., 2003), and glaucomatous optic neuropathy, or glaucoma (Ward et al., 2014; Weitlauf et al., 2014). Glaucoma is the leading cause of irreversible blindness (Quigley and Broman, 2006), involving sensitivity to intraocular pressure (IOP) that stresses retinal ganglion cell (RGC) axons as they form the optic nerve (Calkins, 2012). Many RGCs express TRPV1 channels (Jo et al., 2017; Lakk et al., 2018), localizing to dendrites, unmyelinated axon segment, and cell body, where it increases with short-term elevations in IOP (Sappington et al., 2009, 2015; Weitlauf et al., 2014) but is negligible within the optic nerve itself (Choi et al., 2015). Even so, *Trpv1^–/–^* accelerates optic nerve degeneration with elevated IOP and increases the depolarization necessary for RGCs to produce action potentials (Ward et al., 2014; Weitlauf et al., 2014). To better understand the early stages of this acceleration, we investigated how *Trpv1^–/–^* influences physiological signaling along the optic nerve with short-term elevations in IOP. We found that enhanced excitability in *Trpv1^–/–^* optic nerve was associated with longer axonal nodes of Ranvier with greater levels of the voltage-gated sodium channel, NaV1.6. These results suggest a role for TRPV1 in native tissue to regulate NaV in response to disease-relevant stressors. The absence of this tuning in *Trpv1^–/–^* mice suggests that accelerated axonopathy could arise from excessive excitation even as elevated IOP stresses match available metabolic resources in the optic projection to the brain (Baltan et al., 2010; Calkins, 2012).

## Materials and Methods

### Animal Experiments

Adult male *Trpv1^–/–^* (B6.129 × 1-Trpv1^TM 1Jul^/J) mice (1.5–2 months old) were obtained from The Jackson Laboratory, whereas the appropriate wild-type (WT) background strain C57Bl/6 mice were purchased from Charles River Laboratories (male, 1.5–2 months old). The *Trpv1^–/–^* mice have a targeted mutation causing a non-functional truncated form of TRPV1 (Caterina et al., 2000; Ren et al., 2019; Stanford et al., 2019). *Trpv1^–/–^* animals were genotyped prior to performing experiments, following our protocol (Ward et al., 2014; Weitlauf et al., 2014; Sappington et al., 2015) using primers recommended by the vendor. The mutant forward primer was TAA AGC GCA TGC TCC AGA CT compared with the WT forward primer of TGG CTC ATA TTT GCC TTC AG. The common primer was CAG CCC TAG GAG TTG ATG GA. DNA gel electrophoresis of *Trpv1^–/–^* animals showed a single band at 176 bp, indicative of truncated TRPV1 (Caterina et al., 2000; Ren et al., 2019; Stanford et al., 2019), whereas WT showed a single band at 289 bp indicative of the native protein. We verified this pattern in each animal utilized.

Mice were maintained in a 12 h light/dark cycles, and animals were allowed water and standard rodent chow as desired. All animal experiments were approved by The Vanderbilt University Medical Center Institutional Animal Care and Use Committee. Baseline IOP was measured bilaterally in anesthetized (2.5% isoflurane) mice using Tono-Pen XL (Medtronic Solan) for 1–2 days prior to experimental manipulation. Baseline IOP measurements were averaged (day 0). After baseline IOP measurements, unilateral elevation of IOP was induced by injecting 1.5 μl of 15 μm polystyrene microbeads (Invitrogen) into the anterior chamber; the fellow eye received an equal volume of sterile saline to serve as control. We measured IOP 2–3 times per week for 2 weeks as described previously (Crish et al., 2010; Weitlauf et al., 2014; Risner et al., 2018).

### Optic Nerve Compound Action Potential Electrophysiology

Animals were euthanized by cervical dislocation and decapitated. The skull was cut along the sagittal suture and removed, and the optic nerves were sectioned from the brain. Optic nerves were cut at the optic chiasm and posterior to the optic nerve head, and nerves were placed in carbogen-saturated (95% O_2_, 5% CO_2_) ice-cold (4°C) artificial cerebrospinal fluid (aCSF) for 30 min (Wang et al., 2012). The aCSF contained (in mM/L) 124 NaCl, 3 KCl, 2 CaCl_2_, 2 MgCl_2_, 1.25 NaH_2_PO_4_, 23 NaHCO_3_, and 10 glucose (Baltan et al., 2010). The pH of the aCSF was 7.4.

Optic nerves were incubated in ice-cold aCSF to slow metabolism because we recorded from optic nerves one at a time. The first nerve recorded from (saline- or microbead-injected eyes) was alternated daily to avoid any possible order effects. After incubation, one optic nerve was transferred into a physiological chamber (Model PH1, Warner Instruments) and continually perfused at a rate of 2 mL/min using a peristaltic pump (Model 7518, Masterflex) and maintained at 35°C (Model TC-344C, Warner Instruments). Optic nerves adjusted to physiological conditions for 30 min prior to recording. After adjustment to physiological conditions, the rostral end of the optic nerve was positioned into a bipolar recording suction electrode (Model 573040, A-M Systems), and the caudal end of the optic nerve was positioned into a custom-made bipolar stimulating suction electrode. The syringe section of each electrode was attached to separate micromanipulators (Model MM33, WPI) to allow fine positioning of the electrodes. The electrode section of the suction electrodes was fabricated from borosilicate glass (Model TW150-4, WPI) that was heat-pulled (Model P2000, Sutter Instruments) to form an average opening of ∼350 μm in diameter. The stimulating electrode contained a Ag wire, and the recording pipette contained a Ag/AgCl wire; both pipettes were filled with aCSF.

Evoked potentials were bandpass filtered (0.0001–10 kHz), amplified (100 × gain, DAM-60, WPI), digitized (Digidata 1440A, Molecular Devices), and sampled at 50 kHz (Clampex 10.6, Molecular Devices). Afterward, we measured the resistance between the nerve and recording pipette by stimulating the nerve with 10-μs 100-μA pulses at a minimum of three positions along the optic nerve and measuring the compound action potential (CAP) (Model ISO-STIM 01-DPI, NPI). The resistance of the optic nerve and pipette at each spatial position along the nerve was computed using Ohm’s law.

Current-evoked CAPs were obtained for at least three spatial positions along each optic nerve. Thus, at each spatial position, the resistance between the recording pipette and nerve was unique. We then plotted the resultant CAP area obtained at each spatial position as a function of resistance. Then, we obtained the slope of the linear regression of these data. The slope of the data represents an approximation of the current-induced voltage output of the nerve (Stys et al., 1991).

In a subset of experiments, CAPs were evoked with brief, 10 μs, square pulses, ranging from 10 to 200 V, every 30 s until a maximal response was produced. Maximal response was defined by the peak of the CAP. Once we determined the voltage required to produce a maximum response, we challenged optic nerve excitability by bath application of 300 and 600 nM of 4,9-anhydrotetrodotoxin (aTTX; Alomone Labs) or 100 nM of iodoresiniferatoxin (IRTX; Tocris). After 5 min of drug application, an evoked CAP was obtained using the max-response stimulus previously determined under normal bath conditions. To assess excitability within the optic nerve, we computed the percent decrease or percent of baseline of the evoked CAP based on before and after drug responses.

At the end of each recording session, optic nerves were placed in 4% paraformaldehyde overnight at −4°C. Afterward, we placed nerves on slides, imaged nerves on a microscope slide micrometer, and quantified length and width using the “segmented line” tool in ImageJ [Version 1.51i, National Institutes of Health (NIH)]. The average optic nerve width for WT and *Trpv1^–/–^* mice was 0.329 ± 0.004 and 0.333 ± 0.003 mm, respectively. There was no difference in optic nerve length between genotypes (*p* = 0.45) or between experimental condition (*p* = 0.56).

### Optic Nerve Immunohistochemistry, Imaging, and Analysis

For optic nerve sections, mice were first perfused with phosphate-buffered saline (PBS) followed by 4% paraformaldehyde. Optic nerves were placed separately into optimal cutting temperature (OCT) compound (Fisher Scientific). Optic nerves were sectioned longitudinally every 7 μm, taking care to keep the nerves as flat as possible. Sections were first blocked with 5% normal donkey serum for 2 h and then incubated in primary antibodies for 3 days at 4°C with gentle shaking. Primary antibodies used for optic nerve sections were mouse-contactin-associated protein 1 (Caspr1, 1:300, Millipore) and rabbit-NaV1.6 (1:200, Alomone). Confocal micrographs of all sections were acquired using an Olympus FV1000 inverted microscope with 100 × objective and 2 × zoom.

Optic nerve node–paranode complexes were assessed using similar methods as Arancibia-Cárcamo et al. (2017). To determine the length of the node and paranode segments for each node–paranode complex, the following analysis was performed for each complex using a series of custom-written MATLAB functions: First, the most prominent trough of the Caspr1 staining intensity profile was noted, and the location of its minimum point identified. Next, the most prominent peak to both the right and left of this minimum point was identified. These maxima were averaged, and half of the average value was used to define a threshold intensity value to distinguish node and paranode segments. For each of the two identified peaks, the contiguous region surrounding the peak and above the threshold was considered paranode, whereas the region between the two paranode segments and under the threshold was considered node. The length of these segments and their average staining intensity (Caspr1 for paranode and NaV1.6 for node) were calculated.

All data are presented as mean ± SEM. Graphs were made using Sigma Plot Version 14 (Systat, San Jose, CA, United States). Statistical analyses were performed using Sigma Plot and Matlab (R2019a, Natick, MA, United States). Parametric statistics were performed (*t*-tests, ANOVAs) if data passed normality and equal variance tests; otherwise, we performed non-parametric statistics (Mann–Whitney, ANOVA on ranks).

## Results

### *Trpv1^–/–^* Following Short-Term Intraocular Pressure Elevation Increases Optic Nerve Excitability

**FIGURE 1:**
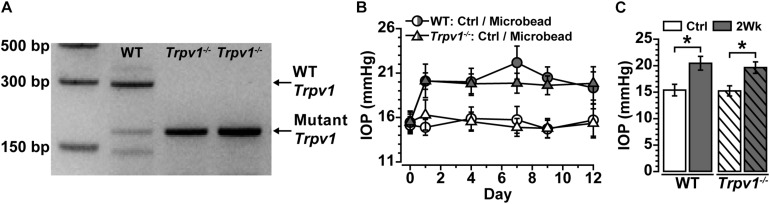
**(A)** Genotype confirmation shows the band for wild-type (WT) *Trpv1* at 289 bp (lane 1, C57 background strain) vs. the 176 bp mutant *Trpv1* (lanes 2 and 3). **(B)** Intraocular pressure (IOP) for WT and *Trpv1^–/–^* mice following unilateral injection of microbeads (vs. saline injection control, Ctrl) was similar between genotypes. **(C)** IOP significantly increased in WT (33%) and *Trpv1^–/–^* (29%) eyes compared with their respective saline-injected control eyes (WT: *p < 0.01, *Trpv1^–/–^*: *p < 0.01). Statistics: Independent samples *t*-tests. *n* = 16 (WT Ctrl), 16 (WT 2Wk), 15 (*Trpv1^–/–^* Ctrl), and 15 (*Trpv1^–/–^* 2Wk).

Following our protocol for conformational genotyping (Ward et al., 2014; Weitlauf et al., 2014; Sappington et al., 2015), *Trpv1^–/–^* mice showed a single product band at 176 bp, indicative of a non-functional truncated form of *Trpv1* (Caterina et al., 2000; Ren et al., 2019; Stanford et al., 2019), whereas WT C57 mice had a prominent band at 289 bp characteristic of the native protein (Figure 1A).

Recently, we discovered that short-term (2 weeks) elevations in IOP enhance excitability in multiple types of RGCs and their axons (Risner et al., 2018). Following the same procedure for unilateral microbead injection, IOP significantly increased for the 2-week duration of the experiment for both WT and *Trpv1^–/–^* mice (Figure 1B). In WT mice, IOP increased by 33% (20.5 ± 1.3 mmHg) compared with saline-injected eyes (15.4 ± 1.1 mmHg, ^∗^*p* < 0.01). Similarly, in *Trpv1^–/–^* mice, IOP increased by 29% in microbead-injected eyes (19.6 ± 1.1 mmHg) relative to saline controls (15.2 ± 1.0 mmHg, *p* < 0.01, Figure 1C). Genotype had no influence on IOP for either saline- or microbead-injected eyes (*p* ≥ 0.96).

**FIGURE 2:**
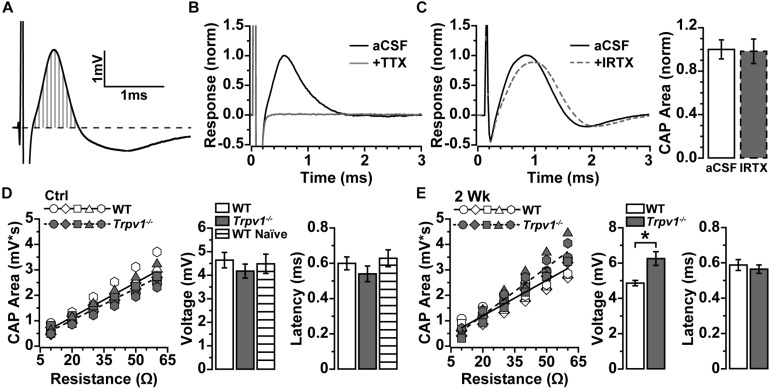
**(A)** Compound action potential (CAP) area measured as integral (vertical gray lines) above baseline (dashed line) for current-evoked voltage changes over time. **(B)** Example of CAP from wild-type (WT) naïve optic nerve in artificial cerebral spinal fluid (aCSF) and with 1 μM of tetrodotoxin (TTX) added, which eliminated the CAP. **(C)** Example CAP from WT naïve optic nerve before and after bath application of 100 nM of iodoresiniferatoxin (IRTX) (left), which did not influence area when normalized to aCSF (*p* = 0.34, *n* = 5). **(D)** Integrated CAP calculated as in **(A)** increases with nerve resistance for individual WT (*n* = 7) and *Trpv1^–/–^* (*n* = 5) nerves from control eyes. Slope of best-fitting regression line indicates CAP voltage (right), which did not differ between WT, *Trpv1^–/–^*, and WT naïve (*n* = 4; *p* = 0.62). Latency too did not differ (*p* = 0.40). **(E)** Integrated CAP for individual WT (*n* = 7) and *Trpv1^–/–^* (*n* = 5) nerves following 2 weeks of elevated IOP (left). For *Trpv1^–/–^* nerves, elevated IOP increased slope of best-fitting line compared with that of corresponding control (CAP voltage, right; *p = 0.001). Latency did not differ for either WT or *Trpv1^–/–^* nerves compared with control nerves (*p* = 0.59). Statistics: **(C,E)** independent samples *t*-tests; **(D)** one-way ANOVA.

To determine whether IOP modulates electrical activity in the myelinated optic nerve as it does for the retina, we measured the current-evoked CAP (Baltan et al., 2010). Optic nerve CAP typically demonstrated a single voltage peak following depolarizing current stimulation (Figure 2A), which could be eliminated by blocking voltage-gated sodium channels with tetrodotoxin (TTX; 1 μM; Figure 2B). In the retina, RGC excitability can be modulated directly by TRPV1 activation and inhibition (Weitlauf et al., 2014). This is not so for optic nerve. Application of the TRPV1-specific antagonist IRTX at sub-micromolar concentrations known to inhibit TRPV1 (Wahl et al., 2001) did not significantly affect the evoked CAP for naïve WT optic nerve (*p* = 0.91, Figure 2C).

Resistance to stimulating current varies with axon density and diameter, extra-axonal space and glia, and positioning of the recording electrode, all of which alter the measured response (Stys et al., 1991). To compare optic nerve CAP between animals more accurately, we obtained multiple measurements while varying the positioning of the recording electrode. As resistance increased, so too did the integral of the CAP response (Figures 2D,E), with the slope of the best-fitting line yielding a more precise measure of CAP voltage (Stys et al., 1991). In addition, we assessed the amount of time required for axons to conduct action potentials by measuring the response latency as the time from stimulus onset to peak of the CAP. For control nerves, *Trpv1^–/–^* did not influence the CAP voltage (*p* = 0.16) or latency (*p* = 0.40) as compared with WT (Figure 2D). In contrast, following 2 weeks of elevated IOP, *Trpv1^–/–^* significantly increased the CAP voltage relative to control nerves (6.3 ± 0.4 vs. 4.2 ± 0.3 mV; *p* = 0.001) but did not modulate latency. Elevated IOP did not affect the WT CAP voltage or latency as compared with control nerves (Figure 2E).

### *Trpv1^–/–^* Optic Nerve Is Less Sensitive to NaV1.6 Antagonism

**FIGURE 3:**
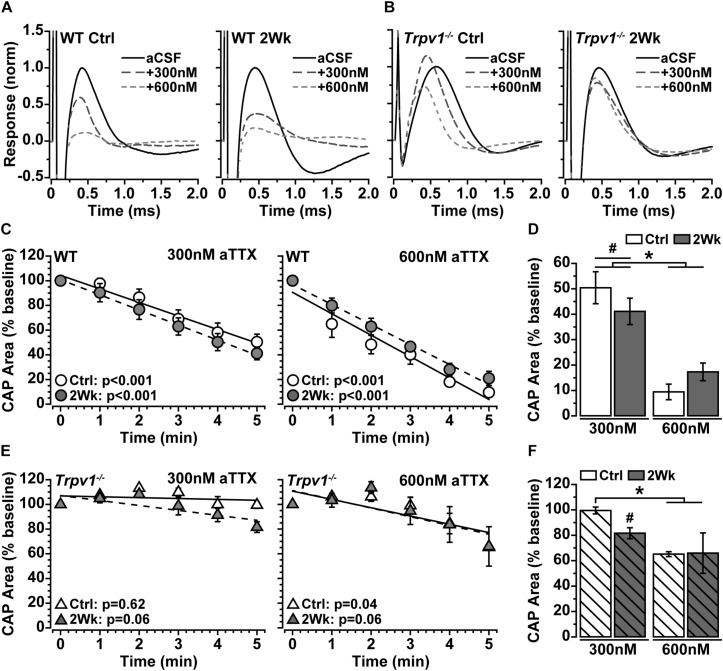
**(A,B)** Example compound action potential (CAP) responses of nerves from Ctrl eyes and following 2 weeks of elevated intraocular pressure (IOP) from wild-type (WT) and *Trpv1^–/–^* mice with bath application of 300 and 600 nM of aTTX. **(C)** Mean WT CAP area for control and 2-week nerves decreases over time following bath application of 300 and 600 nM of aTTX. Individual recordings normalized to corresponding baseline (pre-drug) response. Slopes of best-fitting regression lines indicated significant decline (*p*-values indicated). **(D)** Final CAP area for WT decreases significantly following 300 nM of aTTX for both control (*n* = 7, 50% decrease) and 2-week (*n* = 6, 59% decrease) nerves compared with baseline for each (^#^*p* ≤ 0.03). CAP area decreased further from baseline for control (*n* = 6, 91% decrease) and 2-week nerves (*n* = 6, 83% decrease) following application of 600 nM of aTTX, both significant declines compared with 300 nM (*p < 0.001). **(E)** Mean *Trpv1^–/–^* CAP area following bath application of 300 and 600 nM of aTTX; for slopes of best-fitting regression lines, only control nerves with 600 nM of aTTX showed significant decline (*p*-values indicated). **(F)** Final CAP area for *Trpv1^–/–^* control nerves were minimally affected by 300 nM of aTTX (*n* = 5, 0.5% decrease), whereas area for 2-week nerves declined compared with baseline (*n* = 5, 18% decrease; ^#^*p* = 0.02). Like WT, 600 nM of aTTX caused a greater reduction in CAP area compared with 300 nM for Ctrl nerves (35% decrease; *p = 0.02). Statistics: **(C,E)** linear regressions; **(D,F)**: one-way ANOVAs, Tukey *post-hoc*.

Action potentials are propagated in myelinated nerve by activation of the voltage-gated sodium (NaV) channel 1.6, which densely accumulates within nodes of Ranvier (Craner et al., 2003). Because IRTX did not significantly modulate optic nerve CAP (Figure 2C), we tested whether the increase in *Trpv1^–/–^* optic nerve CAP with elevated IOP (Figure 2E) is due to NaV1.6 activity. We again measured optic nerve CAP following bath application of 300 and 600 nM of aTTX, a selective inhibitor of the NaV1.6 subunit (Hargus et al., 2013). For WT optic nerve, the CAP was suppressed by 300 nM and further reduced by 600 nM of aTTX (Figure 3A). In contrast, the *Trpv1^–/–^* optic nerve CAP appeared relatively insensitive to aTTX of either concentration (Figure 3B). We quantified the influence of aTTX as the percent decrease in CAP area following drug administration, normalized to baseline area for each nerve. In WT nerve, regardless of IOP elevation or aTTX concentration, CAP area declined significantly with time after drug application (Figure 3C), as indicated by the slope of the best-fitting regression line. The CAP for WT control nerves decreased by 50% following 300 nM of aTTX and by 91% following 600 nM of aTTX compared with baseline (Figure 3D). Elevated IOP had little influence for either aTTX concentration, as compared with control nerves (*p* ≥ 0.53). For *Trpv1^–/–^*, aTTX had little influence on CAP over time, with the slope of the best-fitting regression line significantly declining only for 600 nM of aTTX treatment of control nerves (Figure 3E). With 300 nM, only the CAP for 2-week nerves declined compared with baseline, whereas only control nerves declined further with 600 nM compared with treatment with 300 nM (Figure 3F).

### *Trpv1^–/–^* Alters NaV1.6 Density and Node Length With Elevated Intraocular Pressure

**FIGURE 4:**
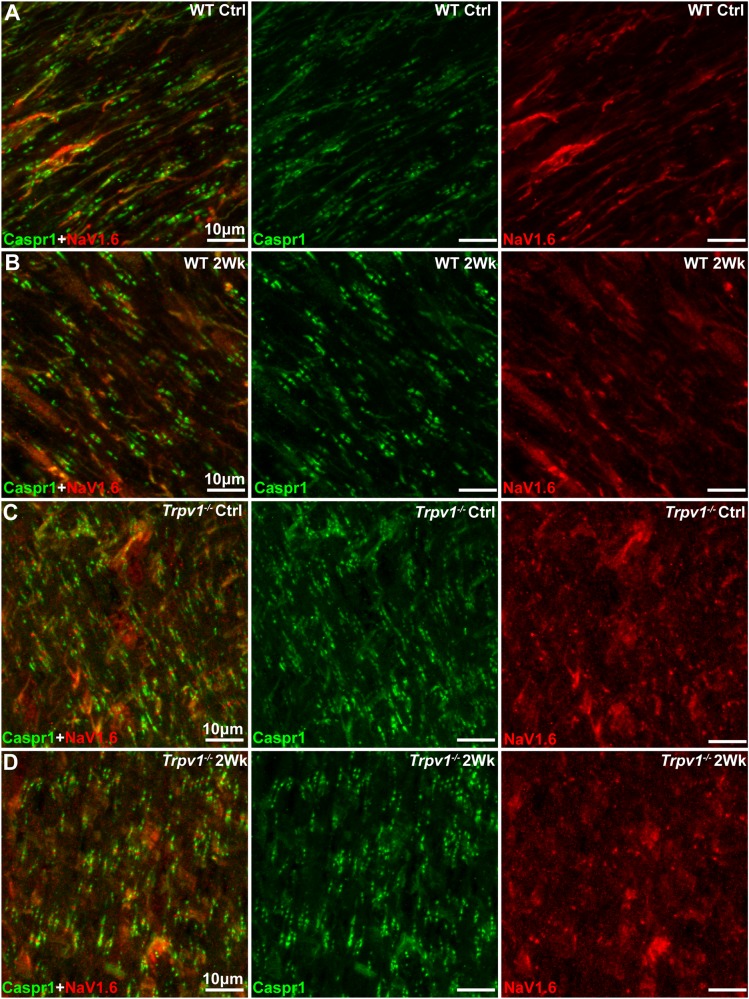
**(A–D)** Representative confocal micrographs of Caspr1 (green) and NaV1.6 (red) immunostaining of longitudinal optic nerve sections from wild-type (WT) **(A,B)** and *Trpv1^–/–^* **(C,D)** mice. Scale bar = 10 μm.

The results in Figure 3 indicate that *Trpv1^–/–^* optic nerve is relatively insensitive to aTTX suppression of NaV1.6 activation than are WT nerves. In the myelinated optic nerve, NaV1.6 localizes to nodes of Ranvier flanked by paranodes defined by the membrane protein Caspr1 (contactin associated protein 1; Craner et al., 2003). Immunolabeling for NaV1.6 and Caspr1 in longitudinal sections confirmed this fundamental configuration in both WT and *Trpv1^–/–^* optic nerves (Figure 4). Compared with WT nerves from control and IOP-stressed eyes (Figures 4A,B), the node–paranode complex appeared smaller in *Trpv1^–/–^* optic nerves with more intense location of NaV1.6 (Figures 4C,D).

**FIGURE 5:**
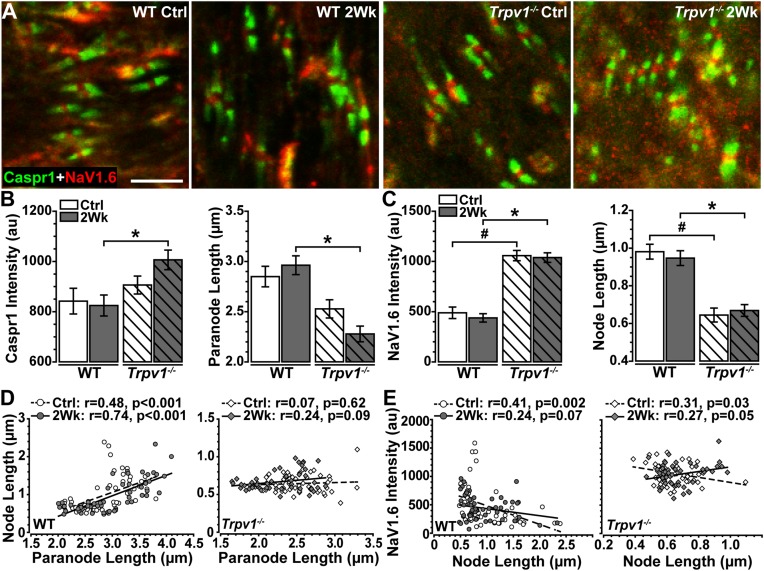
**(A)** High magnification confocal micrographs of longitudinal sections through Ctrl and 2 Wk wild-type (WT) and *Trpv1^–/–^* optic nerves show Caspr1-labeled paranodes (green) flanking NaV1.6 (red) within nodes of Ranvier. **(B)** *Trpv1^–/–^* 2 Wk paranodes contain increased Caspr1 than did WT 2 Wk nerves (left, *p = 0.012) and are shorter (right, *p < 0.001). **(C)** NaV1.6 is higher in nodes of Ranvier of *Trpv1^–/–^* nerves (left) compared with WT Ctrl (^#^*p* < 0.001) and 2 Wk (*p < 0.001) optic nerve (left), although node length is significantly shorter for both *Trpv1^–/–^* Ctrl (^#^*p* < 0.001) and 2 Wk (*p < 0.001) nerves. Elevated intraocular pressure (IOP) had no effect on either measure (*p* ≥ 0.88). **(D)** There is a positive relationship between node and paranode lengths in WT optic nerves (left), however, this relationship is lost in *Trpv1^–/–^* (right) optic nerves. Elevated IOP had no effect on this relationship for WT or *Trpv1^–/–^* optic nerves. **(E)** NaV1.6 intensity decreases as node length increases in WT (left) and *Trpv1^–/–^* (right) control optic nerves. Following IOP elevation, this relationship is lost in WT nerves, whereas the relationship becomes positive in *Trpv1^–/–^* nerves. Scale = 5 μm **(A)**. Statistics: **(B,C)**: one-way ANOVAs, Tukey *post-hoc*; **(D,E)** linear regressions. Total nodes analyzed: WT Ctrl, 3,942; WT 2 Wk, 3,890; *Trpv1^–/–^* Ctrl, 2,024; *Trpv1^–/–^* 2 Wk, 2,191. Five animals per condition. Ten images per animal.

To quantify these apparent differences, we measured paranode and node length and intensity of Caspr1 and NaV1.6 localization within well-defined paranode–node complexes (Figure 5A). For WT optic nerve, elevated IOP had no effect on levels of paranodal Caspr1 compared with control (*p* = 0.76) nor on paranode length (*p* = 0.81; Figure 5B). However, for *Trpv1^–/–^* optic nerve, elevated IOP increased Caspr1 significantly compared with that for WT (*p* = 0.012; Figure 5B, left) whereas significantly shortening paranode length compared with that for WT (*p* < 0.001; Figure 5B, right). Within the nodes themselves, NaV1.6 was significantly higher for *Trpv1^–/–^* compared with WT for both control and 2-week nerves (*p* < 0.001; Figure 5C, left). As with Caspr1-labeled paranodes, *Trpv1^–/–^* significantly shortened the nodes compared with WT (*p* < 0.001; Figure 5C, right). Thus, NaV1.6 concentrates at a higher level in truncated paranode–node complexes in *Trpv1^–/–^* optic nerve. We found significant positive correlations between node and paranode length in WT control and 2-week nerves (*p* < 0.001, Figure 5D, left). For *Trpv1^–/–^* optic nerve, there was no correlation (Ctrl, *p* = 0.62; 2Wk, *p* = 0.09, Figure 5D, right). For both WT and *Trpv1^–/–^* control nerves, NaV1.6 intensity decreased significantly with increasing nodal length, so that NaV1.6 was more concentrated in shorter nodes (*p* ≤ 0.03, Figure 5E). However, for *Trpv1^–/–^* nerves with elevated IOP, the relationship was reversed so that NaV1.6 concentrated in *longer* nodes (*p* = 0.05, Figure 5E, right); this was not so for WT nerves (*p* = 0.07). These results suggest that the combined increase in NaV1.6 localization with decreased length of the paranodal complex strengthens the *Trpv1^–/–^* CAP, rendering these nerves far less sensitive to aTTX antagonism (Figure 3). That elevated IOP increases NaV1.6 with increasing node length likely explains the increased CAP for *Trpv1^–/–^* nerves under these conditions.

## Discussion

Previously, we found that *Trpv1^–/–^* accelerates optic nerve axonopathy with elevated IOP, reducing nerve area, axon density, and axon transport to the brain (Ward et al., 2014). The deleterious influence of *Trpv1^–/–^* on nerve health and axon function with IOP-related stress likely can be linked to cationic activity. Here, we sought to determine the impact of *Trpv1^–/–^* with short-term IOP elevation on optic nerve signaling to the brain, using the evoked CAP. As expected (Baltan et al., 2010), the optic nerve CAP demonstrated a single-peaked voltage inflection in response to depolarizing current that was eliminated by application of the voltage-gated sodium channel antagonist, TTX (Figures 2A,B), underscoring the dependence of CAP on these channels. Under control conditions, in the absence of IOP-related stress, the CAP for WT and *Trpv1^–/–^* optic nerves was identical, and naïve CAP was unaffected by specific pharmacological antagonism of TRPV1 (Figures 2C,D).

Our key physiological result is that modest, short-term IOP elevation significantly increases *Trpv1^–/–^* optic nerve CAP. On the surface, this finding is paradoxical. We have recently shown TRPV1 expression and RGC excitability concurrently increase following 2 weeks of elevated IOP (Weitlauf et al., 2014). In that study, *Trpv1^–/–^* eliminated the stress-related enhancement of RGC excitability, and *Trpv1^–/–^* RGCs required larger depolarizing currents to generate action potentials with elevated IOP. On the basis of this collective evidence, one would expect IOP elevation to reduce *Trpv1^–/–^* optic nerve CAPs. How then do we explain our results? *Trpv1^–/–^* nerves were relatively impervious to NaV1.6 antagonism by aTTX, which suppressed the WT CAP (Figure 3). This difference accompanies shorter nodes of Ranvier with far greater NaV1.6 localization in *Trpv1^–/–^* but not WT nerves (Figures 4, 5). In fact, NaV1.6 in WT optic nerve nodes is unaltered following up to 5 weeks of microbead-induced IOP elevation (Smith et al., 2018). This novel finding suggests that TRPV1, which is typically associated with presynaptic potentiation of glutamatergic action (Marinelli et al., 2003; Medvedeva et al., 2008), can also tune channel expression within axons – even though localization of TRPV1 in the optic nerve head is negligible (Choi et al., 2015).

Our data show that *Trpv1^–/–^* causes a compensatory aggregation of NaV1.6 protein expression within nodes of Ranvier and a significant decrease in nodal length (Figure 5C). This may serve as a cautionary note that genetic excision of a single gene, *Trpv1* in this case, can lead to unexpected effects on neuronal structure and expression levels of other channels. Here, we observed that *Trpv1^–/–^* led to increased NaV1.6 expression, which conferred greater resistance to the NaV1.6 antagonist, aTTX (Figure 3). The general observation that overexpression of a drug target correlates with a higher resistance to inhibition is a fundamental assumption for drug target identification. This assumption is often true when inhibition of the target only reduces target activity. However, if inhibition of the target also catalyzes harmful downstream effects, drug efficacy cannot be predicted (Palmer and Kishony, 2014). Although it is unknown if inhibition of NaV1.6 by aTTX impacts off-target sites, here, we find that for WT control nerves, 300 nM of aTTX caused a 50% reduction of the CAP and 600 nM of aTTX decreased the CAP near 100%, suggesting that NaV1.6 resistance to aTTX is linear (Figures 3D,F).

Finally, we found that *Trpv1^–/–^* with elevated IOP causes a modest but significant shift in the relationship between NaV1.6 expression and node length, where NaV1.6 accumulates more in longer nodes (Figure 5E). Interestingly, others have found that increased nodal length and ectopic expression of NaV1.6 in aged optic nerves are related to larger CAP despite decreased levels of ATP (Stahon et al., 2016). We previously found that elevated IOP in *Trpv1^–/–^* mice accelerates ATP-dependent anterograde axon transport deficits and optic nerve axon degeneration (Ward et al., 2014). Ultimately, our results indicate that IOP-related stress, like aging, requires a redistribution of energy resources at the expense of axon transport to preserve voltage-dependent axon signaling. In the absence of TRPV1, this demand is increased, further taxing a vulnerable system. Thus, in glaucoma and other age-related neurodegenerative diseases, TRPV1 may reconfigure NaV expression in neurons under stress to normalize excitability to existing metabolic resources.

## Data Availability Statement

The datasets generated for this study are available on request to the corresponding author.

## Ethics Statement

The animal study was reviewed and approved by Vanderbilt University IACUC.

## Author Contributions

NM, MR, and DC designed research and wrote the manuscript. NM and MR performed research. NM, MR, VV, and DC analyzed data.

## Conflict of Interest

The authors declare that the research was conducted in the absence of any commercial or financial relationships that could be construed as a potential conflict of interest.

## References

[B1] Arancibia-Cárcamo I. L. Ford M. C. Cossell L. Ishida K. Tohyama K. Attwell D. (2017). Node of Ranvier length as a potential regulator of myelinated axon conduction speed. *eLife* 6:e23329. 10.7554/eLife.23329 28130923 PMC5313058

[B2] Balleza-Tapia H. Crux S. Andrade-Talavera Y. Dolz-Gaiton P. Papadia D. Chen G. (2018). TrpV1 receptor activation rescues neuronal function and network gamma oscillations from Abeta-induced impairment in mouse hippocampus in vitro. *eLife* 7:e37703. 10.7554/eLife.37703 30417826 PMC6281315

[B3] Baltan S. Inman D. M. Danilov C. A. Morrison R. S. Calkins D. J. Horner P. J. (2010). Metabolic vulnerability disposes retinal ganglion cell axons to dysfunction in a model of glaucomatous degeneration. *J. Neurosci.* 30 5644–5652. 10.1523/JNEUROSCI.5956-09.2010 20410117 PMC2884009

[B4] Bolcskei K. Helyes Z. Szabo A. Sandor K. Elekes K. Nemeth J. (2005). Investigation of the role of TRPV1 receptors in acute and chronic nociceptive processes using gene-deficient mice. *Pain* 117 368–376. 10.1016/j.pain.2005.06.024 16150543

[B5] Calkins D. J. (2012). Critical pathogenic events underlying progression of neurodegeneration in glaucoma. *Prog. Retin. Eye Res.* 31 702–719. 10.1016/j.preteyeres.2012.07.001 22871543 PMC3472111

[B6] Caterina M. J. Leffler A. Malmberg A. B. Martin W. J. Trafton J. Petersen-Zeitz K. R. (2000). Impaired nociception and pain sensation in mice lacking the capsaicin receptor. *Science* 288 306–313. 10.1126/science.288.5464.306 10764638

[B7] Caterina M. J. Schumacher M. A. Tominaga M. Rosen T. A. Levine J. D. Julius D. (1997). The capsaicin receptor: a heat-activated ion channel in the pain pathway. *Nature* 389 816–824. 10.1038/39807 9349813

[B8] Choi H. J. Sun D. Jakobs T. C. (2015). Astrocytes in the optic nerve head express putative mechanosensitive channels. *Mol. Vis.* 21 749–766. 26236150 PMC4502055

[B9] Chung Y. C. Baek J. Y. Kim S. R. (2017). Capsaicin prevents degeneration of dopamine neurons by inhibiting glial activation and oxidative stress in the MPTP model of Parkinson’s disease. *Exp. Mol. Med.* 49:e298. 10.1038/emm.2016.159 28255166 PMC5382554

[B10] Craner M. J. Lo A. C. Black J. A. Waxman S. G. (2003). Abnormal sodium channel distribution in optic nerve axons in a model of inflammatory demyelination. *Brain* 126 1552–1561. 10.1093/brain/awg153 12805113

[B11] Crish S. D. Sappington R. M. Inman D. M. Horner P. J. Calkins D. J. (2010). Distal axonopathy with structural persistence in glaucomatous neurodegeneration. *Proc. Natl., Acad. Sci. U.S.A.* 107 5196–5201. 10.1073/pnas.0913141107 20194762 PMC2841892

[B12] Cristino L. de Petrocellis L. Pryce G. Baker D. Guglielmotti V. Di Marzo V. (2006). Immunohistochemical localization of cannabinoid type 1 and vanilloid transient receptor potential vanilloid type 1 receptors in the mouse brain. *Neuroscience* 139 1405–1415. 10.1016/j.neuroscience.2006.02.074 16603318

[B13] Hargus N. J. Nigam A. Bertram E. H. III Patel M. K. (2013). Evidence for a role of Nav1.6 in facilitating increases in neuronal hyperexcitability during epileptogenesis. *J. Neurophysiol.* 110 1144–1157. 10.1152/jn.00383.2013 23741036 PMC3763090

[B14] Hui K. Liu B. Qin F. (2003). Capsaicin activation of the pain receptor, VR1: multiple open states from both partial and full binding. *Biophys. J.* 84 2957–2968. 10.1016/s0006-3495(03)70022-8 12719227 PMC1302858

[B15] Jayant S. Sharma B. M. Sharma B. (2016). Protective effect of transient receptor potential vanilloid subtype 1 (TRPV1) modulator, against behavioral, biochemical and structural damage in experimental models of Alzheimer’s disease. *Brain Res.* 1642 397–408. 10.1016/j.brainres.2016.04.022 27084583

[B16] Jo A. O. Noel J. M. Lakk M. Yarishkin O. Ryskamp D. A. Shibasaki K. (2017). Mouse retinal ganglion cell signalling is dynamically modulated through parallel anterograde activation of cannabinoid and vanilloid pathways. *J. Physiol.* 595 6499–6516. 10.1113/JP274562 28766743 PMC5638913

[B17] Lakk M. Young D. Baumann J. M. Jo A. O. Hu H. Krizaj D. (2018). Polymodal TRPV1 and TRPV4 sensors colocalize but do not functionally interact in a subpopulation of mouse retinal ganglion cells. *Front. Cell Neurosci.* 12:353. 10.3389/fncel.2018.00353 30386208 PMC6198093

[B18] Lastres-Becker I. de Miguel R. De Petrocellis L. Makriyannis A. Di Marzo V. Fernandez-Ruiz J. (2003). Compounds acting at the endocannabinoid and/or endovanilloid systems reduce hyperkinesia in a rat model of Huntington’s disease. *J. Neurochem.* 84 1097–1109. 10.1046/j.1471-4159.2003.01595.x 12603833

[B19] Marinelli S. Di Marzo V. Berretta N. Matias I. Maccarrone M. Bernardi G. (2003). Presynaptic facilitation of glutamatergic synapses to dopaminergic neurons of the rat substantia nigra by endogenous stimulation of vanilloid receptors. *J. Neurosci.* 23 3136–3144. 10.1523/jneurosci.23-08-03136.2003 12716921 PMC6742307

[B20] Medvedeva Y. V. Kim M. S. Usachev Y. M. (2008). Mechanisms of prolonged presynaptic Ca2+ signaling and glutamate release induced by TRPV1 activation in rat sensory neurons. *J. Neurosci.* 28 5295–5311. 10.1523/JNEUROSCI.4810-07.2008 18480286 PMC2694046

[B21] Mezey E. Toth Z. E. Cortright D. N. Arzubi M. K. Krause J. E. Elde R. (2000). Distribution of mRNA for vanilloid receptor subtype 1 (VR1), and VR1-like immunoreactivity, in the central nervous system of the rat and human. *Proc. Natl.Acad. Sci. U.S.A.* 97 3655–3660. 10.1073/pnas.97.7.3655 10725386 PMC16295

[B22] Morgese M. G. Cassano T. Cuomo V. Giuffrida A. (2007). Anti-dyskinetic effects of cannabinoids in a rat model of Parkinson’s disease: role of CB(1) and TRPV1 receptors. *Exp. Neurol.* 208 110–119. 10.1016/j.expneurol.2007.07.021 17900568 PMC2128772

[B23] Nam J. H. Park E. S. Won S. Y. Lee Y. A. Kim K. I. Jeong J. Y. (2015). TRPV1 on astrocytes rescues nigral dopamine neurons in Parkinson’s disease via CNTF. *Brain* 138 3610–3622. 10.1093/brain/awv297 26490328 PMC4840550

[B24] Palmer A. C. Kishony R. (2014). Opposing effects of target overexpression reveal drug mechanisms. *Nat. Commun.* 5:4296. 10.1038/ncomms5296 24980690 PMC4408919

[B25] Patapoutian A. Tate S. Woolf C. J. (2009). Transient receptor potential channels: targeting pain at the source. *Nat. Rev. Drug Discov.* 8 55–68. 10.1038/nrd2757 19116627 PMC2755576

[B26] Quigley H. A. Broman A. T. (2006). The number of people with glaucoma worldwide in 2010 and 2020. *Br. J. Ophthalmol.* 90 262–267. 10.1136/bjo.2005.081224 16488940 PMC1856963

[B27] Ren X. Roessler A. E. Lynch T. L. T. Haar L. Mallick F. Lui Y. (2019). Cardioprotection via the skin: nociceptor-induced conditioning against cardiac MI in the NIC of time. *Am. J. Physiol.* 316 H543–H553. 10.1152/ajpheart.00094.2018 30575436 PMC6415820

[B28] Risner M. L. Pasini S. Cooper M. L. Lambert W. S. Calkins D. J. (2018). Axogenic mechanism enhances retinal ganglion cell excitability during early progression in glaucoma. *Proc. Natl.Acad. Sci. U.S.A.* 115 E2393–E2402. 10.1073/pnas.1714888115 29463759 PMC5877940

[B29] Roberts J. C. Davis J. B. Benham C. D. (2004). [3H]Resiniferatoxin autoradiography in the CNS of wild-type and TRPV1 null mice defines TRPV1 (VR-1) protein distribution. *Brain Res.* 995 176–183. 10.1016/j.brainres.2003.10.001 14672807

[B30] Sappington R. M. Sidorova T. Long D. J. Calkins D. J. (2009). TRPV1: contribution to retinal ganglion cell apoptosis and increased intracellular Ca2+ with exposure to hydrostatic pressure. *Investi. Ophthalmol. Vis. Sci.* 50 717–728. 10.1167/iovs.08-2321 18952924 PMC3549616

[B31] Sappington R. M. Sidorova T. Ward N. J. Chakravarthy R. Ho K. W. Calkins D. J. (2015). Activation of transient receptor potential vanilloid-1 (TRPV1) influences how retinal ganglion cell neurons respond to pressure-related stress. *Channels* 9 102–113. 10.1080/19336950.2015.1009272 25713995 PMC4594535

[B32] Simone D. A. Baumann T. K. LaMotte R. H. (1989). Dose-dependent pain and mechanical hyperalgesia in humans after intradermal injection of capsaicin. *Pain* 38 99–107. 10.1016/0304-3959(89)90079-1 2780068

[B33] Smith M. A. Plyler E. S. Dengler-Crish C. M. Meier J. Crish S. D. (2018). Nodes of ranvier in glaucoma. *Neuroscience* 390 104–118. 10.1016/j.neuroscience.2018.08.016 30149050

[B34] Stahon K. E. Bastian C. Griffith S. Kidd G. J. Brunet S. Baltan S. (2016). Age-related changes in axonal and mitochondrial ultrastructure and function in white matter. *J. Neurosci.* 36 9990–10001. 10.1523/JNEUROSCI.1316-16.2016 27683897 PMC5039264

[B35] Stanford K. R. Hadley S. H. Barannikov I. Ajmo J. M. Bahia P. K. Taylor-Clark T. E. (2019). Antimycin A-induced mitochondrial dysfunction activates vagal sensory neurons via ROS-dependent activation of TRPA1 and ROS-independent activation of TRPV1. *Brain Res.* 1715 94–105. 10.1016/j.brainres.2019.03.029 30914247 PMC6500470

[B36] Stys P. K. Ransom B. R. Waxman S. G. (1991). Compound action potential of nerve recorded by suction electrode: a theoretical and experimental analysis. *Brain Res.* 546 18–32. 10.1016/0006-8993(91)91154-s 1855148

[B37] Wahl P. Foged C. Tullin S. Thomsen C. (2001). Iodo-resiniferatoxin, a new potent vanilloid receptor antagonist. *Mol. Pharmacol.* 59 9–15. 10.1124/mol.59.1.9 11125018

[B38] Wang Q. Vlkolinsky R. Xie M. Obenaus A. Song S. K. (2012). Diffusion tensor imaging detected optic nerve injury correlates with decreased compound action potentials after murine retinal ischemia. *Investig. Ophthalmol. Vis. Sci.* 53 136–142. 10.1167/iovs.11-7908 22159023 PMC3292354

[B39] Ward N. J. Ho K. W. Lambert W. S. Weitlauf C. Calkins D. J. (2014). Absence of transient receptor potential vanilloid-1 accelerates stress-induced axonopathy in the optic projection. *J. Neurosci.* 34 3161–3170. 10.1523/JNEUROSCI.4089-13.2014 24573275 PMC3935081

[B40] Weitlauf C. Ward N. J. Lambert W. S. Sidorova T. N. Ho K. W. Sappington R. M. (2014). Short-term increases in transient receptor potential vanilloid-1 mediate stress-induced enhancement of neuronal excitation. *J. Neurosci.* 34 15369–15381. 10.1523/JNEUROSCI.3424-14.2014 25392504 PMC4228139

